# Positive Effect of a Pea–Clam Two-Peptide Composite on Hypertension and Organ Protection in Spontaneously Hypertensive Rats

**DOI:** 10.3390/nu14194069

**Published:** 2022-09-30

**Authors:** Xiaopeng Sun, Min Wang, Chuanjin Xu, Shanglong Wang, Li Li, Shengcan Zou, Jia Yu, Yuxi Wei

**Affiliations:** 1 College of Life Sciences, Qingdao University, Qingdao 266071, China; 2 Chenland Nutritionals, Inc., Invine, CA 92614, USA; 3 The Affiliated Hospital of Medical College, Qingdao University, Qingdao 266071, China

**Keywords:** pea-clam two-peptide composite, antihypertension, spontaneously hypertensive rats, RAS system, organ protection

## Abstract

In the present study, we prepared pea peptides with high angiotensin-converting enzyme (ACE) inhibitory activity in vitro using an enzymatic hydrolysis of pea protein and compounded them with clam peptides to obtain a pea-clam double peptide. The effects of the two-peptide composite and pea peptides on hypertension and the damage-repair of corresponding organs were studied in spontaneously hypertensive rats (SHRs). We found that both pea peptides and the two-peptide composite significantly reduced the blood pressure upon a single or long-term intragastric administration, with the two-peptide composite being more effective. Mechanistically, we found that the two-peptide composite could regulate the renal renin-angiotensin system (RAS), rebalance gut microbial dysbiosis, decrease renal and myocardial fibrosis, and improve renal and cardiac function and vascular remodeling. Additionally, hippocampal lesions caused by hypertension were also eliminated after two-peptide composite administration. Our research provides a scientific basis for the use of this two-peptide composite as a safe antihypertension ingredient in functional foods.

## 1. Introduction

Epidemiological studies have shown that hypertension is a common chronic disease that is the most unneglectable contributor to the global burden of disease [1]. Its complications, such as cardiovascular disease, stroke, and chronic kidney disease, cause nearly 10 million deaths each year and are a leading risk factor for premature deaths worldwide [2,3]. According to statistical data from 2017 [4], the global population with hypertension accounted for about 35%, while only nearly 60% of patients received treatment, and half of them could not effectively control their blood pressure. These hypertensive patients may need to take long-term or even lifelong antihypertensive medications.

The induction of hypertension is related to a complex interplay of environmental, hereditary, and other internal or external factors, including increased cardiac output or systemic vascular resistance, excessive activation of sympathetic nervous systems, vascular remodeling, excessive release of endothelins, enhancement of the renin–angiotensin system (RAS), and abnormal glomerular capillary filtration function [3,5,6]. The RAS is a major factor controlling changes in blood pressure, which is regulated by renin and mediated by the vital stress hormone angiotensin II (AngII) to lead to powerful vasoconstrictive actions [7,8]. The main function of the angiotensin-converting enzyme (ACE) is to catalyze the conversion of angiotensin I (AngI) into AngII, and it is considered to be the core enzyme in the RAS [9,10]. Therefore, ACE inhibitors (ACEIs) are increasingly becoming the main hypertension treatment drugs [6]. Initial ACEIs were separated and extracted from snake venom, and their potency was unsatisfactory. Then, synthetic ACEIs such as ramipril, lisinopril, perindopril, enalapril, and captopril were developed and have been successively used clinically as antihypertensive drugs, especially for the treatment of renal hypertension (high renin/high aldosterone) [11,12,13]. However, the clinical application of these synthetic ACEIs also brings certain toxicity and side effects, as they may cause neutropenia, renal toxicity, angioedema, asthma, and hepatotoxicity [14,15,16]. Patients with hypertension may need long-term or even lifelong medication, which means the side effects of synthetic ACEI should not be ignored. Therefore, more efficient and safer ACEIs from natural food ingredients are of great significance for hypertension treatment. Previous studies have reported that natural ACEIs are mainly derived from milk and some animals and plants [17], such as active peptides in various fermented milk products [18], the protein hydrolysate of pearl oyster (Pinctada fucata martensii) [19], and soy protein hydrolysates [20]. Moreover, plant-derived ACEIs, especially those from legumes, have obtained more attention than the other two. ACEIs have been obtained by soybean fermentation and the enzymatic hydrolysis of pea, pigeonpea, chickpea, or adzuki bean [21,22,23,24]. Legumes exhibit more advantages in industrial production, offering wider sources, cheaper costs, and more convenient transport and storage. To balance the nutritive and functional properties of foods, the combined use of animal and plant proteins has been reported to present a more effective biological activity. For example, soy–whey protein can enhance exercise performance and improve exercise-induced fatigue in rats [25].

In previous studies, we explored the ACE inhibitory activity and reducing blood pressure effect of fermented clam peptides [26]. In this study, we hypothesized that the combination of legume peptides and animal peptides from two natural food ingredients would present excellent effects in the treatment of hypertension, using a simpler method with nutritional and industrial applications. Therefore, we prepared pea peptides with ACEI activity through the enzymolysis of pea protein and proportionally compounded it with clam peptides prepared by enzymatic hydrolysis. The intention of this study was to assess the hypotensive effect of a pea–clam two-peptide composite and positive effects on corresponding organs in spontaneously hypertensive rats (SHRs), and identify its partial mechanism.

## 2. Materials and Methods

### 2.1. Materials

Pea peptides were prepared from the crude pea protein remaining from the production of pea starch by compound protease hydrolysis, and the ACE inhibition rate of pea peptides in vitro was determined according to the available literature method [27]. Clams (Ruditapes philippinarum) were from the local aquatic market in Hongdao (Qingdao, China). We hydrolyzed them with compound protease, and clam peptides were obtained after impurity removal as described previously [28]. The contents of pea and clam peptides were measured by the biuret method, and the average molecular weight of the pea and clam peptides was measured by high-performance liquid chromatography (HPLC) [10]. The two-peptide composite was prepared by mixing pea peptides and clam peptides in a 2:3 (W:W) ratio. Captopril was purchased from Shandong Xinhua Pharmaceutical Co., Ltd. (Shandong, China), and compound proteinase was provided by Shanghai Yuanye Bio-Technology Co., Ltd. (Shanghai, China).

### 2.2. Animals

Twelve-week-old female SHRs (n = 30) and Wistar Kyoto rats (WKY) (n = 6) were purchased from Vital River Experimental Technology Co., Ltd. (SCXK 2021-0006, Beijing, China). Our experiment was authorized by the Animal Care and Use Ethics Committee of Qingdao University and the experimental procedures strictly abide by the ethical welfare requirements of Animal of Qingdao University (Animal Ethics Certificate No.20210902WKY6SHR241010043).

### 2.3. Animal Experiment Scheme Design

The experimental rats were fed strictly according to the corresponding requirements and were given a standard rat diet and adequate drinking water. After an adaptive phase (7 days), the SHRs and WKY rats were randomly assigned to 6 groups (n = 5) and daily administered the following, intragastrically: (1) WKY group, 1 mL·kg^−1^·d^−1^ saline; (2) SHR group, same as WKY group; (3) SHR-C group, 8 mg·kg^−1^·d^−1^ captopril; (4) SHR-DP group, 450 mg·kg^−1^·d^−1^ two-peptide composite; and (5) SHR-PP group, 180 mg·kg^−1^·d^−1^ pea peptides. The doses of two-peptide composite and pea peptides were calculated based on recommended human doses (Based on recommended doses of similar antihypertensive functional food peptides on the market in China). Samples of the prepared solution were delivered into the stomachs of rats using a syringe with an intragastric needle.

At 0, 2, 4, 8, 12, and 24 h after the initial administration, we used the tail-cuff method of BP2010A (Softron Beijing Biotechnology Co., Ltd., Beijing, China) to measure rat blood pressure. Then, blood pressure was measured every Saturday for four weeks during the long-term intragastric administration.

After 4 weeks of administration, all rats were weighed and sacrificed under anesthesia. The collected blood samples were centrifuged to isolate serum. Kidney, heart, and liver were separated and weighed, while portions of the thoracic aorta, whole brain, kidney, and heart tissue were soaked and fixed in 4% paraformaldehyde stationary liquid (Wuhan Servicebio Technology Co., Ltd., Wuhan, China) for subsequent sectioning and pathological staining analysis.

### 2.4. Serum Biochemical Analysis

Serum markers related to renal and cardiac function were measured by an automatic biochemical analyzer (Chemray240 and Chemray800 of Shenzhen Ruiduo Life Analysis Science Co., Ltd., Shenzhen, China). Angiotensin-converting enzyme (ACE), angiotensin-converting enzyme 2 (ACE2), angiotensin 1-7 (Ang1-7), angiotensin II (AngII), glutathione-s-transferase (GST), and homocysteine (Hcy) were detected by their corresponding ELISA kits. Rat angiotensin-converting enzyme ELISA Kit (JM-01619R1), Rat angiotensin-converting enzyme 2 ELISA Kit (JM-02021R1), Rat angiotensin 1-7 ELISA Kit (JM-02020R1), Rat angiotensin II ELISA Kit (JM-01618R1), and Rat glutathione-s-transferase ELISA Kit (JM-02215R1) were provided by Jingmei Biotech. Co. (Shenzhen, China), and Rat homocysteine ELISA Kit (ml403902) was provided by Shanghai Enzyme-linked Biotechnology Co., Ltd. (Shanghai, China).

### 2.5. Pathological Section and Histopathological Analysis

The fixed tissues were sectioned in paraffin according to the method in the literature [29], and pathology was analyzed by pathological staining. CaseViewer 2.4 and Image-Pro Plus 6.0 were used to measure and calculate the wall cross-sectional area (WCSR), lumen cross-sectional area (LCSR), inner diameter (ID), and outer diameter (WD) of the thoracic aorta. Image-Pro Plus6.0 was used to measure and calculate the ratio of collagen fiber to muscle fiber area and collagen volume fraction (CVF) in the kidney, myocardium, and thoracic aorta.

### 2.6. Intestinal Microbial Diversity Analysis

Feces were collected one day before the rats were sacrificed. All stool samples were tested for intestinal microbe using 16S rRNA sequencing by Shanghai Personal Biotechnology Co., Ltd. (Shanghai, China). The QIAamp DNA Fecal Mini Kit (No. 51504) produced by QIAGEN China (Shanghai, China) Co., Ltd. was used to extract microbial genomic DNA from fecal samples of SHRs and WKY rats. Paired-end sequencing of the fecal microbial genomic DNA was conducted by the Illumina NovaSeq platform (Illumina, San Diego, CA, USA), and then taxonomic composition analysis, alpha diversity, beta diversity, and Venn diagrams were accomplished with tools from the Genescloud data analysis platform (https://www.genescloud.cn (accessed on 15 November 2021)) from Shanghai Personal Biotechnology Co., Ltd. [30].

### 2.7. Statistical Data Analysis

The full text data are presented as the mean ± SD. After the normality test, significant differences were analyzed by one-way ANOVA, which was followed by Tukey’s test. Values of *p* < 0.05 were considered to denote statistical significance, while *p* < 0.01 was considered to denote highly significant differences. All data were analyzed using SPSS 19.0.

## 3. Results

### 3.1. Peptide Content, Molecular Weight, and ACE Inhibitory Activity of Pea and Clam Peptides

The peptide content of pea peptides after enzymatic hydrolysis of the pea protein was 91.6%, with an average molecular weight of 494 Da, of which the molecular weights of <500 Da, 500–1000 Da, 1000–2000 Da and >2000 Da accounted for 55.28%, 28.80%, 12.68%, and 3.1% of the peptides, respectively. The clam hydrolysate, after removal of impurities, had a peptide content of 75.6%, with an average molecular weight of 396 Da. Peptides with a molecular weight of less than 500 Da accounted for 73.94% of the peptides, while those molecular weights of 500–1000 Da, 1000–2000 Da and >2000 Da accounted for 19.81%, 5.18%, and 0.77%, respectively. Both pea and clam peptides had high ACE inhibitory activity in vitro. The ACE inhibitory activity of pea peptides was 50.30%, while that of clam peptides was 61.7%. The results suggested that both pea and clam peptides present high ACE inhibition rates in vitro.

### 3.2. Effects of Pea Peptide and Two-Peptide Composite on Blood Pressure of SHRs and WKY Rats

**Figure 1:**
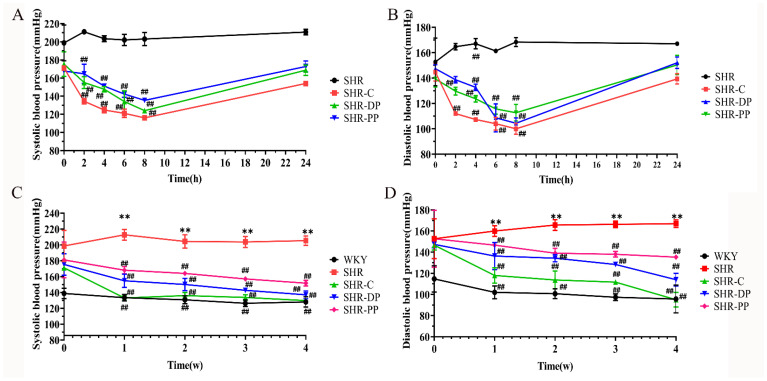
Changes of blood pressure of all rats after single and long-term drug intervention. (**A**,**B**) SBP and DBP of rats in each group after the single drug intervention; (**C**,**D**) SBP and DBP of rats in each group after the long-term drug intervention. The data in the figure are expressed as mean ± SEM. ** *p* < 0.01, represents the difference from the WKY group, respectively; ## *p* < 0.01, represents the difference from the SHR group, respectively.

Although pea and clam peptides showed high inhibitory activity against ACE in vitro, their antihypertensive influence after digestive absorption through the gastrointestinal tract in vivo has not been explicitly researched. Therefore, we monitored changes in SBP (systolic blood pressure) and DBP (diastolic blood pressure) in SHRs and WKY rats after the single and long-term administration of pea peptides and two-peptide composite. As shown in Figure 1A,B, at 2 h after an initial administration, the blood pressure of SHRs decreased significantly in the three drug intervention groups (*p* < 0.01). Moreover, after the first administration, the SBP and DBP of each treatment group decreased continuously to 8 h. Notably, at the 8th hour after intragastric administration, the SBP and DBP of the SHR-PP group decreased to 135.33 ± 2.05 mmHg (*p* < 0.01) and 112.67 ± 5.31 mmHg (*p* < 0.01), while those of the SHR-DP group decreased to 124.00 ± 2.45 mmHg (*p* < 0.01) and 104.33 ± 3.40 mmHg (*p* < 0.01), respectively. The results indicated that the short-term hypotensive effect of the SHR-DP group was stronger than that of the SHR-PP group.

After the single intragastric administration experiment, rats were continuously administered pea peptides or the two-peptide composite for 4 weeks to monitor their long-term antihypertensive influence. Figure 1C,D show that the blood pressure before intervention in SHRs was significantly higher than that in WKY rats (*p* < 0.01). During the long-term monitoring of change in hypertension for 4 weeks, the blood pressure in the SHR group continuously increased, with SBP increasing from 198.67 ± 16.05 mmHg to 205.33 ± 4.78 mmHg and DBP from 152.67 ± 15.37 mmHg to 167.00 ± 2.94 mmHg. During treatment, the SBP and DBP decreased of the SHR-C, SHR-PP, and SHR-DP groups, which lasted until the end of the experiment, and finally, they showed a substantial decrease compared with those in the SHR group (*p* < 0.01). Specifically, compared with the SHR group, the maximum decrease in SBP and DBP in the SHR-PP group were 63.67 ± 2.49 mmHg (*p* < 0.01) and 31.67 ± 2.62 mmHg (*p* < 0.01), respectively, while those in the SHR-DP group were 68.33 ± 8.73 mmHg (*p* < 0.01) and 53.00 ± 3.74 mmHg (*p* < 0.01), respectively. Therefore, the two-peptide composite and pea peptides showed significant antihypertensive effects during both single and chronic interventions in vivo. Additionally, the antihypertensive effect of the two-peptide composite was stronger than that of single-component pea peptides.

### 3.3. Effect of Pea Peptides and Two-Peptide Composite on Kidney and the RAS in SHRs and WKY Rats

**Figure 2:**
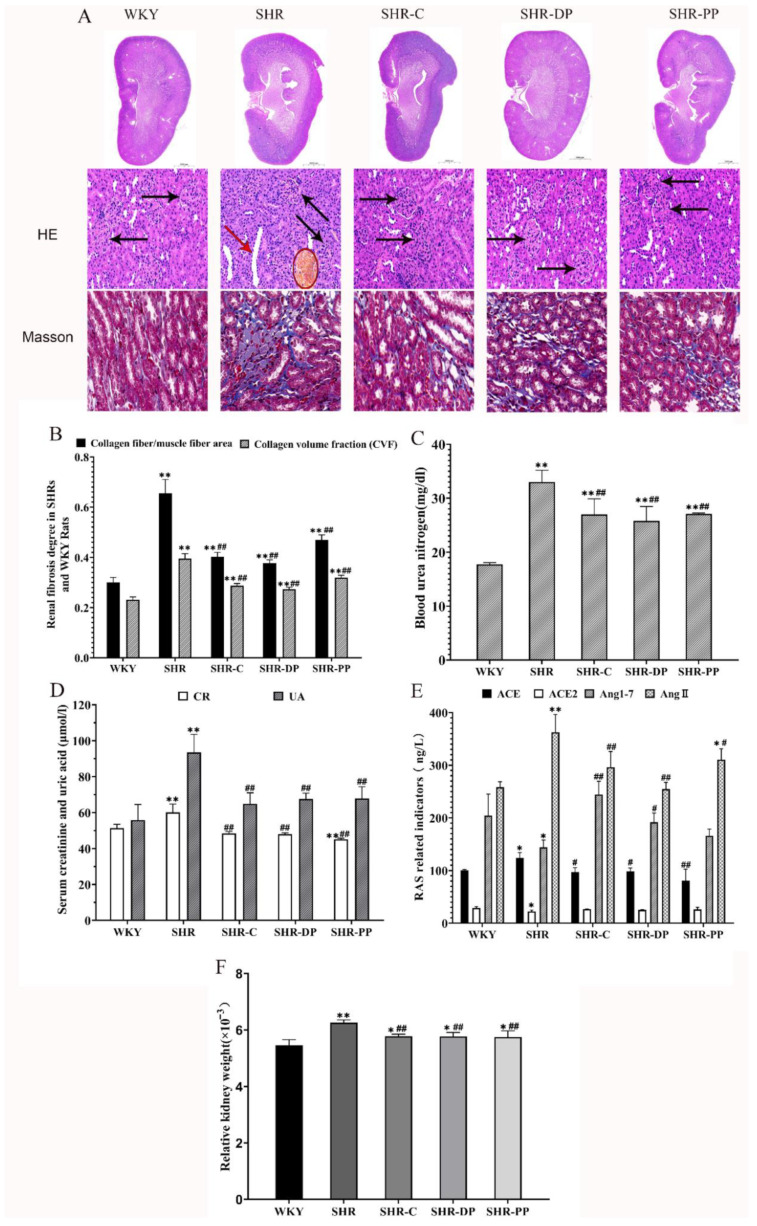
The influence of renal RAS system and renal function and the analysis of renal pathology of rats in each group after pea peptide and two-peptide composite intervention. (**A**) The representative micrographs of rat renal tissues with the HE staining (0.7× and 30×) and Masson staining (63×), glomerulus is represented by black arrow, renal tubule is represented by red arrow and renal interstitial congestion is represented by circle; (**B**) Degree of renal fibrosis in rats of each group; (**C**,**D**) Serum concentration of renal function-related markers (BUN, CR and UA); (**E**) Serum concentration of RAS system markers (ACE, ACE2, AngII, ANG(1-7)); (**F**) The relative organ weight of renal. The data in the figure are expressed as mean ± SEM. * *p* < 0.05, ** *p* < 0.01, represents the difference from the WKY group, respectively; # *p* < 0.05, ## *p* < 0.01, represents the difference from the SHR group, respectively.

Figure 2A shows representative micrographs of rat kidney tissues as a result of HE staining. No obvious renal structure abnormality was found in the WKY group, while the kidneys of the SHR group were deformed, with glomerular atrophy, cystic dilatation of renal tubules, and interstitial hyperemia [31]. As shown by the arrows in Figure 2A, these renal pathological changes were alleviated after intervention by captopril, pea peptides, and the two-peptide composite, with the effect of the two-peptide composite being significantly higher than that of pea peptides. The Masson’s trichrome staining scan results in Figure 2A displayed that the degree of fibrosis in the part of renal medulla in the SHR group was more severe than that in the WKY group. Meanwhile, the kidney collagen fiber area (S1)/muscle fiber area (S) and collagen volume fraction (CVF) in Figure 2B of the SHR group were very increased and much higher than those in the SHR group (*p* < 0.01). After the intervention, renal S1/S and CVF were significantly restored (*p* < 0.01), suggesting that the extent of renal fibrosis was reduced, and the two-peptide composite exhibited a better therapeutic effect on chronic renal lesions caused by hypertension compared to pea peptides and captopril.

As hypertension can cause kidney damage, serum blood urea nitrogen (BUN), creatinine (CR), uric acid (UA), and other indicators highly related to renal function were employed to evaluate the effect of pea peptides and the two-peptide composite on the renal function of SHRs. The concentrations of BUN, CR, and UA in serum are shown in Figure 2C,D, and those in the SHR group were significantly higher than those in the WKY group (*p* < 0.01). After the intervention, the contents of BUN, CR, and UA of SHRs in the SHR-PP and SHR-DP groups were significantly decreased (*p* < 0.01), and both groups showed similar CR levels to the WKY group. These results suggested that pea peptides and two-peptide composite treatment could attenuate the renal damage caused by hypertension.

Moreover, we detected the levels of angiotensin-converting enzyme (ACE), angiotensin-converting enzyme 2 (ACE2), angiotensin 1-7 (Ang1-7), and angiotensin II (AngII) in serum to explore the effect of pea peptides and the two-peptide composite on renal RAS (Figure 2E). We found that compared with the WKY group, the serum contents of ACE and AngII in the SHR group were significantly increased (*p* < 0.05, *p* < 0.01), while those of ACE2 and ANG1-7 were significantly decreased (*p* < 0.05, *p* < 0.01). After pea peptides and two-peptide composite intervention, these four indexes were improved. ACE, Ang(1-7), and AngII of the SHR-DP group were overtly different from those in the SHR group (*p* < 0.05 and *p* < 0.01), while only ACE and AngII of the SHR-PP group were significantly different from those in the SHR group (*p* < 0.05 and *p* < 0.01, respectively). These results suggest that pea peptides and the two-peptide composite can rebalance RAS signaling to regulate blood pressure by down-regulating the ACE-AngII-AT1 axis and up-regulating the ACE2-Ang(1-7)-Mas axis.

Additionally, we measured the rat kidney weight and calculated the relative organ weight (ratio of organ weight/body weight as the visceral coefficient) to evaluate organ weight change (Figure 2F). The relative weight of kidneys in the SHR group was significantly higher than that in the WKY group (*p* < 0.01). After treatment, the relative kidney weight was recovered in both the SHR-DP and SHR-PP groups (*p* < 0.01). We found that the trends in relative kidney weight coefficient changes were consistent with those of the experimental results above. The results demonstrated that the two-peptide composite and pea peptides can reduce kidney damage caused by hypertension.

### 3.4. Effect of Pea Peptides and the Two-Peptide Composite on SHR and WKY Rat Vascular Remodeling

**Figure 3:**
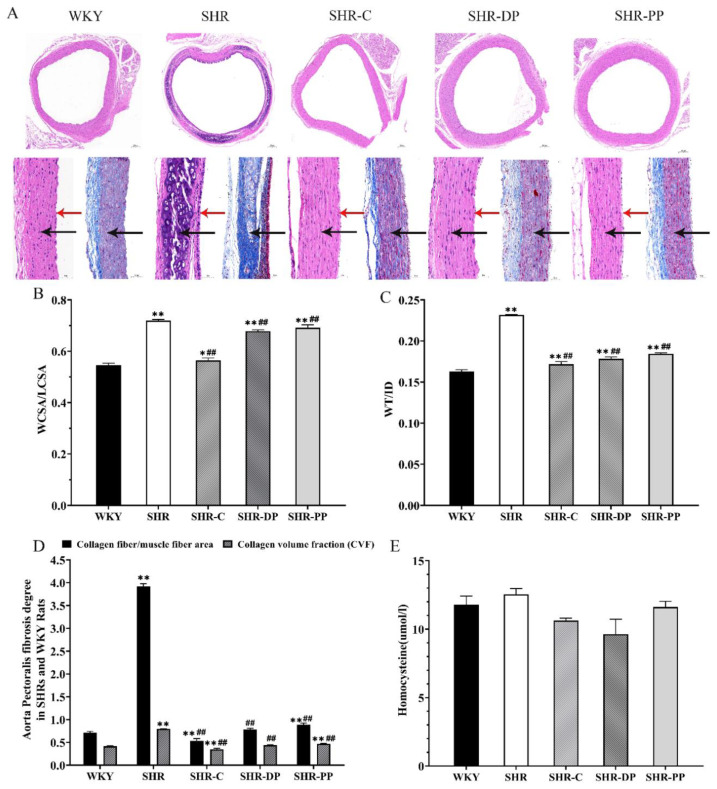
The influence of thoracic aortic vascular remodeling, fibrosis and the concentration of Hcy in the serum of rats in each group after pea peptide and two-peptide composite intervention. (**A**) The representative micrographs of rat thoracic aorta with the HE staining (4.5× and 30×) and Masson staining (20×), the tunica media is represented by a black arrow, endangium is represented by a red arrow; (**B**,**C**) Characterization parameters of vascular remodeling, WCSA/LCSA and WT/ID; (**D**) Degree of thoracic aorta fibrosis in rats of each group; (**E**) Serum concentration of Hcy. The data in the figure are expressed as mean ± SEM. * *p* < 0.05, ** *p* < 0.01, represents the difference from the WKY group, respectively; ## *p* < 0.01, represents the difference from the SHR group, respectively.

Figure 3A exhibits the representative micrographs of rat arcus aortae as a result of HE staining. The vascular morphology of the SHR group was visibly abnormal, with vascular wall thickening, intima rough, and a trend of atherosclerosis. As shown by the arrows in Figure 3A, these arcus aortae pathological changes were significantly alleviated after intervention by captopril, pea peptides, and the two-peptide composite. In Figure 3B,C, the characterization parameters of the thoracic aorta vascular remodeling (WT/ID and WCSR/LCSR) of the SHR group were very significantly increased (*p* < 0.01) compared to the WKY group. Correspondingly, the two parameters in SHR rats were decreased by captopril, pea peptides, and the two-peptide composite (all *p* < 0.01), and their effect on the SHR-DP group was slightly better than on the SHR-PP group. The results of Masson trichrome staining in Figure 3A showed serious fibrosis of the arcus aortae in the SHR group compared with the WKY group. Meanwhile, the arcus aortae collagen fiber area (S1)/muscle fiber area (S) and the collagen volume fraction (CVF) in the SHR group were also very significantly increased (*p* < 0.01) (Figure 3D). After the intervention, the arcus aortae S1/S and CVF decreased significantly (*p* < 0.01), suggesting that the extent of arcus aortae fibrosis was reduced. These results illustrated that the pea peptides and two-peptide composite prevented the thickening of the wall cross-sectional area and wall thickness, reduced the ratio of WT/ID and WCSR/LCSA, restored the lesions of the thoracic aorta, and reduced vascular remodeling and vascular fibrosis. Additionally, the two-peptide composite exhibited a better effect on the repair of the thoracic aorta compared with pea peptides.

We also measured the level of homocysteine (Hcy) in serum due to its relationship with hypertension. Serum Hcy levels were increased in the SHR group compared with those in the WKY group (Figure 3E). After the intervention, serum Hcy decreased in all experimental groups with a most prominent decrease in the SHR-DP group, suggesting that the two-peptide composite could reduce serum Hcy to ameliorate hypertension.

### 3.5. Effect of Pea Peptides and Two-Peptide Composite on Myocardial Fibrosis and Heart Function in SHRs and WKY Rats

**Figure 4:**
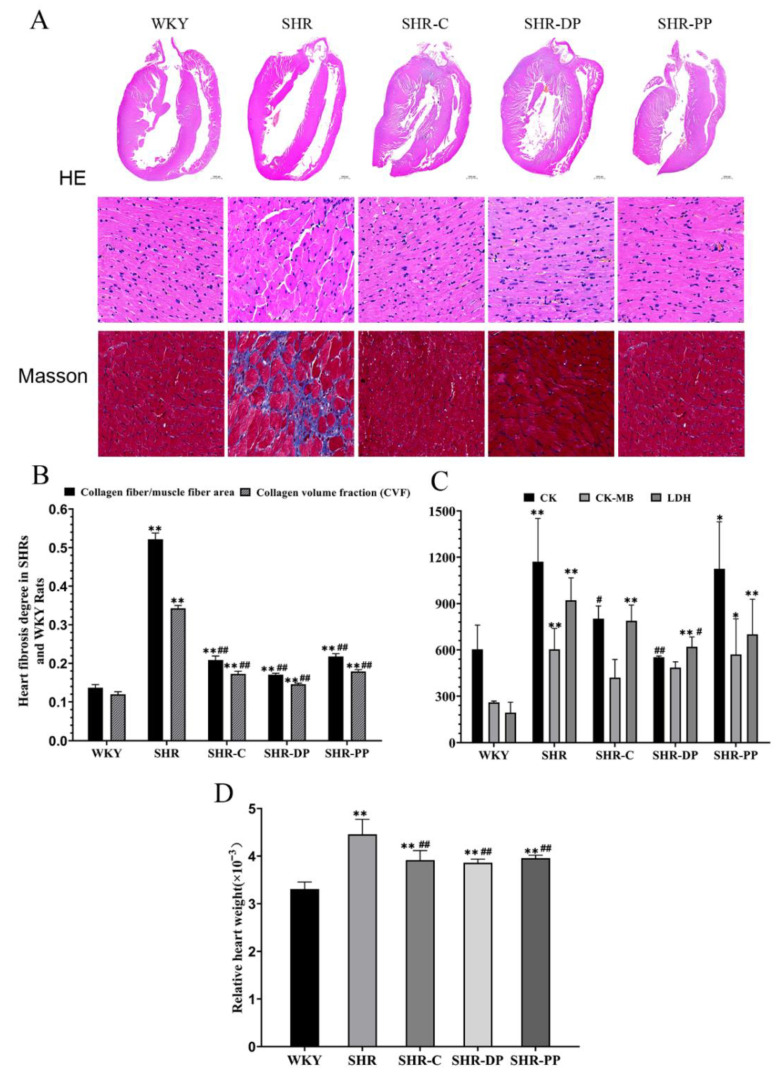
The influence of heart structure and damage to rats in each group after pea peptides and two-peptide composite intervention. (**A**) The representative micrographs of rat heart with the HE staining (0.8× and 40×) and Masson staining (50×); (**B**) Degree of heart fibrosis in rats of each group; (**C**) Serum concentration of heart function-related markers (CK, CK-MB and LDH); (**D**) The relative organ weight of heart. The data in the figure are expressed as mean ± SEM. * *p* < 0.05, ** *p* < 0.01, represents the difference from the WKY group, respectively; # *p* < 0.05, ## *p* < 0.01, represents the difference from the SHR group, respectively.

Hypertension is one of the important factors causing structural changes and myocardial dysfunction [32]. Figure 4A shows representative micrographs of rat heart tissues as a result of HE staining. Cardiomyocytes in the SHR group were hypertrophic with a disordered alignment, while those in the SHR-C, SHR-PP, and SHR-DP groups were recovered and aligned after treatment. Masson trichrome staining scan results (Figure 4A) exhibited partial fibrosis of the myocardium tissues in the SHR group compared with the WKY group. Meanwhile, the myocardial tissue collagen fiber area (S1)/muscle fiber area (S) and the CVF in the SHR group were increased (Figure 4B), with a significant difference between their values in the SHR and WKY groups (both *p* < 0.01). After the intervention, heart S1/S and CVF recovered significantly (*p* < 0.05 and *p* < 0.01), suggesting that both pea peptides and the two-peptide composite could repair myocardial fibrosis caused by hypertension. Additionally, the two-peptide composite exhibited better therapeutic effects than pea peptides and captopril on chronic heart lesions caused by hypertension. 

Moreover, we detected the concentration of creatine kinase, creatine kinase isoenzyme, and lactate dehydrogenase in serum to explore the effect of pea peptides and the two-peptide composite on heart damage (Figure 4C). The concentrations of three indicators of heart function in serum were significantly increased in the SHR group compared with those in the WKY group (*p* < 0.01). The serum concentrations of three indicators of heart function in the SHR-DP group were lower than those in the SHR group, with CK and LDH displaying a significant reduction (*p* < 0.05 and *p* < 0.01), while the serum concentration of these indicators in the SHR-PP group displayed no changes. These results suggested that the two-peptide composite improved the cardiac function decline caused by hypertension, while there was no obvious effect of pea peptides on cardiac function. These results suggested that the two-peptide composite improved the heart damage caused by hypertension, while there was no obvious effect of pea peptides on heart.

Moreover, we weighed the rat hearts and calculated their visceral coefficients (Figure 4D). The relative heart weight in the SHR group was significantly increased (*p* < 0.01) compared to that in the WKY group. After treatment, the relative heart weight was recovered in both SHR-DP and SHR-PP groups (both *p* < 0.01). The results suggested that the two-peptide composite and pea peptides exhibited positive effects on recovering heart damage caused by hypertension.

### 3.6. Effect of Pea Peptides and the Two-Peptide Composite on the Brain of SHRs and WKY Rats

**Figure 5:**
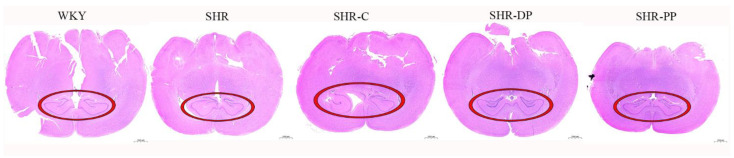
The representative micrographs of rat brain with the HE staining (1×); hippocampus is represented by circle.

Hypertension can cause a series of acute syndromes, among which hypertensive encephalopathy is very serious [33]. To investigate the effects of pea peptides and the two-peptide composite on the brain of SHR rats, the histomorphology of the rat brain was analyzed by HE staining. As indicated by the red circle in Figure 5, the hippocampus of the SHR group was deformed, atrophic, and significantly smaller. However, hippocampal lesions disappeared after the intervention, suggesting that pea peptides and the two-peptide composite may improve brain lesions caused by high blood pressure.

### 3.7. Effect of Pea Peptides and the Two-Peptide Composite on the Liver of SHRs and WKY Rats

**Figure 6:**
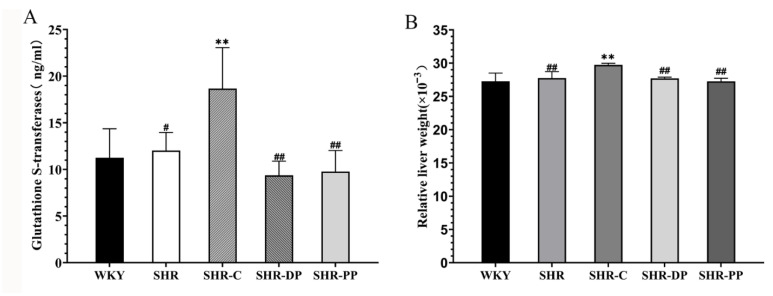
The influence of liver of rats in each group after pea peptide and double peptides intervention. (**A**) Serum concentration of GST; (**B**) The relative organ weight of liver. The data in the figure are expressed as mean ± SEM. ** *p* < 0.01, represents the difference from the WKY group, respectively; # *p* < 0.05, ## *p* < 0.01, represents the difference from the SHR group, respectively.

We determined the level of glutathione thiol transferase (GST) in rat serum to explore whether pea peptides and the two-peptide composite present side effects, as is the case for captopril. The GST serum content in SHRs did not change compared to that of WKY rats (Figure 6A), while the content of serum GST in the SHR-C group was abnormally elevated (*p* < 0.01), which may be related to captopril’s hepatotoxicity. However, the serum GST levels of the SHR-DP and SHR-PP groups did not abnormally increase, and there was a significant difference between them and the SHR-C group (*p* < 0.01). Captopril presented hepatotoxicity, while the two-peptide composite and pea peptides were safer.

Furthermore, we weighed rat livers and calculated their visceral coefficients (Figure 6B). The relative liver weight in the SHR group was significantly increased (*p* < 0.01). After treatment, the abnormal increase in relative liver weight in the SHR-DP and SHR-PP groups was significantly ameliorated (*p* < 0.01) and was closer to that of the WKY group, which was consistent with the above results that the two-peptide composite and pea peptides were safer than captopril.

### 3.8. Effect of Pea Peptides and the Two-Peptide Composite on the Gut Microbiota of SHRs and WKY Rats

**Figure 7:**
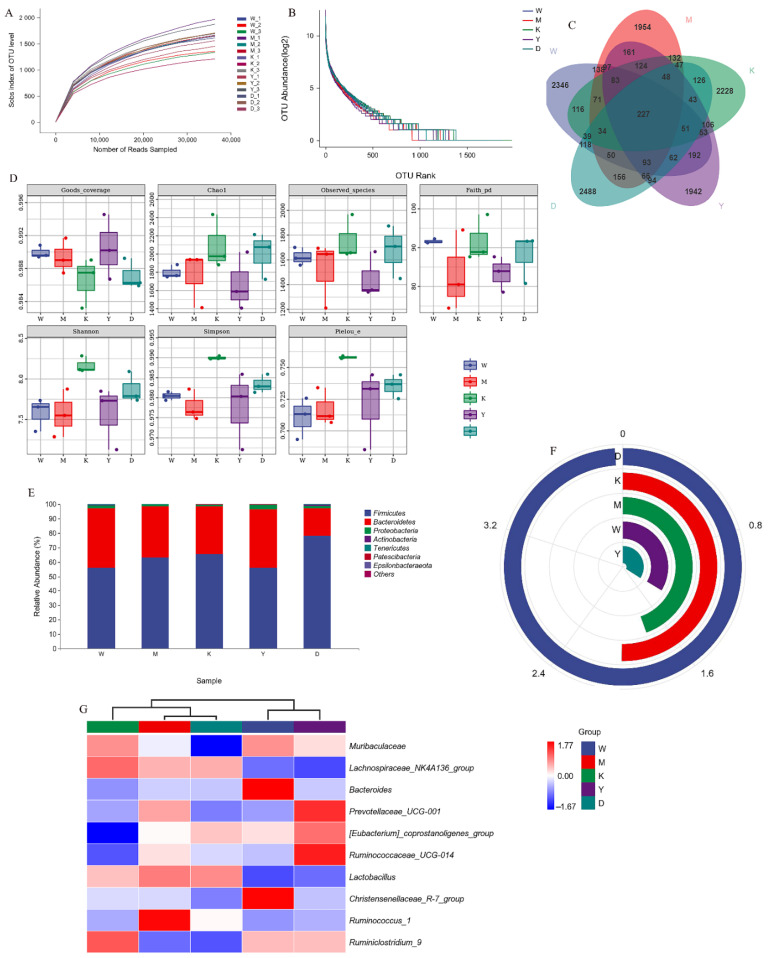
The influence of composition and diversity of gut microorganisms of rats in each group after pea peptide and double peptides intervention. (**A**) Rarefaction curve; (**B**) Rank abundance curve; (**C**) Venn diagram of AVS number; (**D**) α-diversity index; (**E**) The proportion of the top 7 species composition at the phylum level; (**F**) The ratio of F/B in the gut; (**G**) Heatmap showing the normalized Z-score values of microorganism abundances at the genus level. W: WKY, M: SHR, K: SHR-C, Y: SHR-DP, D: SHR-PP.

To explore whether pea peptides and the two-peptide composite can affect blood pressure by interfering with intestinal microorganisms, we studied the structure of intestinal microflora in rat feces. As shown in Figure 7A, with the increased count of optimized sequences, the sparsity curve changed from steep and fast to smooth, indicating that the number of sequencing samples obtained was reasonable. The rank abundance curve in Figure 7B indicated that the curve in the three intervention groups was smoother than that in the SHR group, suggesting that the diversity of the gut microbial structure was increased by pea peptides and two-peptide composite interventions.

Figure 7C presents the results of the Venn diagram-based analysis of amplicon sequence variants (ASVs). The SHR-PP and the SHR-DP groups shared 674 and 858 ASVs with the WKY group, respectively. This showed that the ASV composition of SHRs with two-peptide composite intervention tended to be similar to that of WKY rats. Additionally, the decrease in microbial community diversity is an important feature of intestinal ecological imbalance. The community richness, evenness, and diversity indexes are the evaluation criteria to assess the species diversity of gut microorganisms. The Goods_coverage of each group was over 0.98, suggesting that the sequencing results were reliable and true (Figure 7D). The data of Chao1 and Observed_species indices illustrated that the richness of gut microorganisms was slightly higher in the SHR group compared to that in the WKY group. Although biodiversity relationships mainly rely upon abundance as a representative parameter, evenness also affects community processes and ecosystem functions [34]. The results of the Pielou_e, Faith_pd, Shannon, and Simpson indexes showed that the diversity and uniformity of the SHR group were reduced compared to those of the WKY group, indicating that hypertension affected the rats’ intestinal flora. Gut microbial diversities in the SHR-PP and SHR-DP groups were efficiently improved after intervention compared with that of the SHR group. The SHR-PP group exhibited slightly higher diversity than the SHR-DP group.

## 4. Discussion

Epidemiological studies have shown that hypertension is a common severe chronic disease that is the most unneglectable contributor to the global burden of disease [1]. ACEI is the main high blood pressure drug for patients with renal hypertension (high renin/high aldosterone), while synthetic ACEIs are commonly associated with certain toxicity and side effects [11,12,13,14,15,16]. Thus, more efficient and safer ACEIs from natural food ingredients will be significant in the treatment of hypertension. In this study, we evaluate the antihypertension effect of a pea–clam two-peptide composite in SHRs, exploring its partial molecular mechanism. The results indicated that both pea peptides and the two-peptide composite showed positive antihypertension effects in SHR rats. Additionally, the pea–clam two-peptide composite could also alleviate hypertension-induced corresponding organ damages.

Previous studies have reported that persistently high hypertension often leads to chronic kidney diseases, such as glomerular sclerosis, tubular atrophy, interstitial fibrosis, and even renal failure [35,36,37]. We first investigated the effects of pea peptides and the two-peptide composite on renal histology and function in SHRs. Pathological analysis and relevant calculations showed that the two-peptide composite and pea peptides could reduce the degree of renal disease and fibrosis caused by hypertension, and the effect of the two-peptide composite was more obvious. Meanwhile, the two-peptide composite and pea peptides could significantly reduce the serum concentration of BUN, CR, and UA, demonstrating that they can restore renal function with the effect of the two-peptide composite being more prominent. Previous studies have reported that RAS system disorders play a non-negligible role in the occurrence and progression of hypertension [38,39]. The RAS consists of an ACE-AngII-AT1 axis and an ACE2-Ang(1-7) -Mas axis. In the first axis, ACE promotes the conversion of AngI into AngII, and AngII combines with the Angiotensin type 1 (AT1) receptor to increase blood pressure [40]. In the second axis, ACE2 converts AngI and AngII into Ang(1-9) and Ang(1-7), respectively, while Ang(1-9) can be further converted to Ang(1-7), and Ang(1-7) binds to the G protein-coupled Mas receptor (Mas1) to control blood pressure and prevent heart remodeling [41]. Axis overactivation regulated by ACE and axis inhibition regulated by ACE2 led to elevated blood pressure and kidney damage [42]. Research has shown that the key RAS system components are present in higher concentrations in the kidney [36]. We detected the serum levels of ACE, ACE2, Ang1-7, and AngII to explore the effect of pea peptides and the two-peptide composite on the renal RAS system. The results suggested that pea peptides and the two-peptide composite can rebalance RAS signaling to regulate blood pressure by down-regulating the ACE-AngII-AT1 axis and up-regulating the ACE2-Ang(1-7) -Mas axis, and the effect of the two-peptide composite was more obvious. Moreover, organ weight changes are recognized as sensitive indicators of organ damage, and the ratio of organ weight/body weight is commonly employed as the visceral coefficient to evaluate organ weight change [43]. The kidney index also supports our results, demonstrating that both the two-peptide composite and pea peptides can reduce hypertensive kidney disease, and the effect of the two-peptide composite was more obvious.

Vascular remodeling commonly stands for the pathological change in the structure of resistance vessels caused by hypertension, which leads to increases in systemic vascular resistance and blood pressure, subsequently resulting in a vicious cycle between hypertension and vascular remodeling [44,45]. Typical features of vascular remodeling include vessel wall thickness, lumen shrinkage, and an increased extracellular matrix [46]. We investigated the effects of pea peptides and the two-peptide composite on thoracic aorta vascular remodeling in SHRs. The results of thoracic aorta pathological analysis and calculations show that the two-peptide composite prevented the thickening of the wall cross-sectional area and wall thickness, reduced the values of WT/ID and WCSR/LCSR, repaired the pathological changes of the thoracic aorta, and reduced vascular remodeling and vascular fibrosis. Meanwhile, hyperhomocysteinemia is involved in cardiovascular and neurodegenerative diseases [47]. It has been reported that high levels of Hcy could inhibit vascular dilatation controlled by nitric oxide, induce vascular smooth muscle cell proliferation, change vascular wall elasticity, and induce hypertension [48]. Our results demonstrated that the two-peptide composite can down-regulate serum Hcy, which may be its mechanism of thoracic aorta repair.

The chronic hemodynamic load caused by hypertension can further lead to pathological changes in the myocardial structure, such as cardiomyocyte hypertrophic growth and myocardial interstitial fibrosis [48]. Myocardial fibrosis is the pathological basis of hypertension, which leads to impaired systolic and diastolic function of the heart, ultimately leading to heart failure [49]. The pathological analysis and calculation results of the heart show that the two-peptide composite can eliminate the cardiac lesion caused by hypertension and reduce the degree of cardiac fibrosis. Myocardial fibrosis caused by chronic arterial hypertension can further lead to abnormal cardiac function [49]. In addition, CK, CK-MB, and LDL-H are generally used as the important indicators of hypertension-induced heart injury [50]. In the present study, the two-peptide composite could reduce serum CK, CK-MB, and LDL-L and restore heart function. The heart index also supported our results, demonstrating that both pea peptides and the two-peptide composite can reduce hypertensive heart disease, and the effect of the two-peptide composite was more obvious. In the present study, the two-peptide composite reduced serum CK, CK-MB, and LDL-L and repaired heart damage caused by high blood pressure. Of these, CK-MB is an important marker of heart injury. The heart index also supported our results, demonstrating that both pea peptides and the two-peptide composite can reduce hypertensive heart disease, and the effect of the two-peptide composite is more obvious. However, there are other biomarkers for cardiomyocyte injury and heart function, such as heart-type fatty acid binding protein (HFABP) and left ventricular ejection fraction (LVEF) [51,52]. Therefore, further study could be needed to explore the two-peptide composite’s effect on heart function.

Hypertensive encephalopathy has been reported to be an induced injury, which can lead to hippocampal sclerosis (HS) and refractory temporal lobe epilepsy (TLE) [53]. The results of pathological analysis of brain tissue demonstrated that the two-peptide composite could repair hippocampal lesions caused by hypertension.

Cardiovascular and central nervous system therapeutics are reported to cause chronic drug-induced liver injury (DILI) [54]. Captopril is the most commonly used ACEI class of antihypertensive drug and has been found to present hepatotoxicity and even jaundice or cirrhosis in severe cases [55]. Glutathione transferases are the predominant isoenzymes in hepatocytes, which could detoxify against endogenous and exogenous toxic compounds [56]. Thus, a high concentration of GST in hepatocytes, due to its short half-life, is clinically applied to evaluate liver injury [57]. Changes in serum GST showed that the two-peptide composite and pea peptides were safer than captopril, while the relative liver organ weight also supported this result.

Previous studies have indicated that hypertension can cause an imbalance in gut microbiota structure, down-regulate gut microbiota diversity, and decrease the number of beneficial bacteria [58]. Most studies in recent years have confirmed that gut microbiota dysbiosis was related to the occurrence of hypertension [59]. The two-peptide composite can regulate the structure and improve the diversity of intestinal microbes. Bacteroidetes and Firmicutes are the dominant bacteria in the intestinal tract, and a balance in the ratio of Firmicutes to Bacteroidetes (F/B) is closely related to body health, affecting a variety of physiological functions such as metabolism, maintenance of environmental balance in the gut barrier, and inflammation [60]. An increased F/B ratio, caused by various reasons, is widely recognized as a characteristic feature of gut dysbiosis [58]. Previous studies reported differences in the intestinal microbiota composition between hypertensive and normal rats, identifying increased F/B ratios and reduced microbial diversity in hypertensive rats [61]. In our study, the F/B ratio of SHRs was higher than that of WKY rats, while F/B in the SHR-DP group was lower after the intervention, which suggested that the two-peptide composite may regulate the disorder of intestinal microbiota at a phylum level caused by hypertension. In addition, we found that the F/B ratio in the SHR-PP group was increased, which suggested that the main mechanism of lowering blood pressure for pea peptide may be not closely related to the regulatory effect on gut microbiota. The two-peptide composite could up-regulate Muribaculaceae, [Eubacterium]_coprostanoligenes_group, and Ruminiclostridium_9 and down-regulate Lachnospiraceae_NK4A136_group, Lactobacillus, and Ruminococcus_1 at the generic level. Muribaculaceae, [Eubacterium]_coprostanoligenes_group, and Ruminiclostridium_9 were all reported to be producers of short-chain fatty acids (SCFAs) [62,63,64]. SCFAs may affect blood pressure through the inhibition of histone deacetylases, directly acting on vascular and renal receptors or participating in colonic nerve signaling. In our study, the lactate-producing bacteria in the gut of SHRs proliferated, exceeding the levels found in normal rats, as reported by Verhaar et al. [65]. The Lachnospiraceae_NK4A136_group belongs to Lachnospiraceae, which are intestinal pathogenic bacteria related to intestinal inflammation [66,67]. Ruminococcus_1 is also a pathogenic bacterium, and its produces inflammatory polysaccharides that may directly contribute to gut barrier function breakdown [68]. Gut microbiological analysis showed that the two-peptide composite could improve hypertension by regulating gut microorganism diversity and community structure.

The two-peptide composite showed positive effects in lowering blood pressure and protecting corresponding organs in the four-week in vivo experiment. Since patients with hypertension commonly need long-term or even lifelong medication, further long-term studies are warranted to explore the antihypertensive effects of the two-peptide composite, the protective effect on organs, and possible adverse reactions during long-term administration. In addition, further studies are needed to purify and analyze the sequence of the peptides to evaluate in terms of pharmacology.

## 5. Conclusions

In the present study, we used enzymatic hydrolysis to prepare pea peptides and clam peptides with high ACE inhibitory activity in vitro from two natural food ingredients and compounded them to obtain a two-peptide composite. Both pea peptides and the two-peptide composite showed positive antihypertension effects in SHR rats. We found that the two-peptide composite could reduce blood pressure by regulating renal RAS and rebalancing gut microbial dysbiosis, while it could also eliminate myocardial fibrosis, vascular remodeling, and renal fibrosis, and it could also improve cardiac and renal function as well as hippocampal lesions caused by hypertension. Our research provides a scientific basis for the use of the two-peptide composite as an antihypertension ingredient for functional foods.
